# Pre-treatment levels of circulating free IGF-1 identify NSCLC patients who derive clinical benefit from figitumumab

**DOI:** 10.1038/bjc.2012.497

**Published:** 2012-12-04

**Authors:** A Gualberto, M L Hixon, D D Karp, D Li, S Green, M Dolled-Filhart, L G Paz-Ares, S Novello, J Blakely, C J Langer, M N Pollak

**Retraction to**: *British Journal of Cancer* (2011) **104**, 68–74. doi: 10.1038/sj.bjc.6605972

After a thorough review, the corresponding author, Dr Antonio Gualberto, has concluded that the key results reported in this manuscript are incorrect and cannot be reproduced. As there are no remaining samples that would allow a new analysis, he has therefore recommended the retraction of the manuscript to the Editor-in-Chief of *BJC*.

The co-authors have been informed of this decision.

Dr Gualberto sincerely apologises for any inconvenience this may have caused the readers of the *BJC*.

